# Estimation of Methane Emissions from Slurry Pits below Pig and Cattle Confinements

**DOI:** 10.1371/journal.pone.0160968

**Published:** 2016-08-16

**Authors:** Søren O. Petersen, Anne B. Olsen, Lars Elsgaard, Jin Mi Triolo, Sven G. Sommer

**Affiliations:** 1 Department of Agroecology, Aarhus University, Tjele, Denmark; 2 Institute of Chemical Engineering, Biotechnology and Environmental Technology, University of Southern Denmark, Odense, Denmark; University of Delhi, INDIA

## Abstract

Quantifying in-house emissions of methane (CH_4_) from liquid manure (slurry) is difficult due to high background emissions from enteric processes, yet of great importance for correct estimation of CH_4_ emissions from manure management and effects of treatment technologies such as anaerobic digestion. In this study CH_4_ production rates were determined in 20 pig slurry and 11 cattle slurry samples collected beneath slatted floors on six representative farms; rates were determined within 24 h at temperatures close to the temperature in slurry pits at the time of collection. Methane production rates in pig and cattle slurry differed significantly at 0.030 and 0.011 kg CH_4_ kg^-1^ VS (volatile solids). Current estimates of CH_4_ emissions from pig and cattle manure management correspond to 0.032 and 0.015 kg CH_4_ kg^-1^, respectively, indicating that slurry pits under animal confinements are a significant source. Fractions of degradable volatile solids (VS_d_, kg kg^-1^ VS) were estimated using an aerobic biodegradability assay and total organic C analyses. The VS_d_ in pig and cattle slurry averaged 0.51 and 0.33 kg kg^-1^ VS, and it was estimated that on average 43 and 28% of VS_d_ in fresh excreta from pigs and cattle, respectively, had been lost at the time of sampling. An empirical model of CH_4_ emissions from slurry was reparameterised based on experimental results. A sensitivity analysis indicated that predicted CH_4_ emissions were highly sensitive to uncertainties in the value of lnA of the Arrhenius equation, but much less sensitive to uncertainties in VS_d_ or slurry temperature. A model application indicated that losses of carbon in VS as CO_2_ may be much greater than losses as CH_4_. Implications of these results for the correct estimation of CH_4_ emissions from manure management, and for the mitigation potential of treatments such as anaerobic digestion, are discussed.

## Introduction

In North America and Western Europe around 40% of livestock manure is handled in liquid form [1]. Liquid manure (slurry) represents a mainly anaerobic environment and is a significant source of atmospheric methane (CH_4_), which is the second-largest anthropogenic source of radiative forcing next to carbon dioxide (CO_2_) [2]. Volumes of liquid manure increase in many parts of the world due to intensification of livestock production [3], and thus it becomes increasingly important to determine effects of manure treatment and management on emissions of CH_4_.

In Denmark anaerobic digestion of liquid manure together with energy-rich co-digestates is promoted for bioenergy production, with the ambition that 50% of the total slurry volume should be treated in farm-scale or centralised biogas digesters. The hydrolysis, fermentation and methanogenesis of degradable volatile solids (VS) during anaerobic digestion has the potential to reduce CH_4_ emissions during storage. However, only post-digestion emissions are reduced, and the collection period in slurry pits on the farm is also a source of CH_4_ due to VS degradation [4]. This has implications for the evaluation of climate impacts of anaerobic digestion because CH_4_ emissions from manure management could be underestimated, and the amount of VS available for bioenergy production overestimated, if the composition of VS excreted is used as proxy for the potential bioenergy production. Methods to document CH_4_ emissions and VS loss from slurry pits are therefore urgently needed.

The guidelines of the Intergovernmental Panel on Climate Change [5] only contains a tentative and static method to estimate CH_4_ emissions from slurry pits under animal confinements. It is represented by methane conversion factors (MCFs) that indicate the proportion of the biological CH_4_ production potential emitted. For example, with <30 d storage in slurry pits MCFs of 3 and 30% are recommended for regions with mean annual temperatures below and above 25°C, respectively. Considering the diversity of housing systems, management practices and temperature [6], this approach is clearly inadequate for any detailed assessment of climate impacts.

Quantifying CH_4_ emissions from slurry pits in representative housing systems may be a first step towards an improved methodology for estimating the source strength of slurry pits under animal confinements. Representativeness is defined not only by design parameters but also by manure management, since practices such as emptying routine and cleaning between production cycles can affect in-house emissions [7–9]. Direct measurements of in-house CH_4_ emissions from manure are complicated by the fact that these emissions are not readily separated from those derived from enteric processes [10]. Monteny et al. [4] estimated the contribution from manure to total CH_4_ emissions from housing facilities for dairy cattle and pigs (i.e., not including emissions from outside storage tanks or lagoons) at, respectively, 17–25 and 65–70%. Since direct emissions from livestock may vary also with time of day and stage of a production cycle [11,12], estimation of CH_4_ emissions from manure with a mass balance approach is highly uncertain. An alternative strategy would be to collect manure samples for determination of CH_4_ production rates under controlled conditions.

Attempts have been made to predict CH_4_ emissions from slurry pits. Mechanistic modelling is difficult due to the requirement of data for model parameterisation [13]. A simpler, empirical approach was proposed by Sommer et al. [14] who described algorithms to quantify daily CH_4_ emissions during in-house (and outside) storage, with degradation of manure VS and storage temperature as the main drivers. This model allows for estimation of daily CH_4_ emission and VS loss in slurry pits, and hence could also help estimate the loss of biogas potential. However, Sommer et al. [14] used data from different storage experiments for parameterisation, and until now this empirical model has not been evaluated against experimental data.

The objectives of the study presented here were 1) to estimate CH_4_ emissions from slurry pits on pig and cattle farms delivering slurry for centralised biogas production; and 2) to derive parameters for the model of Sommer et al. [14] based on experimental results, in order to produce a generalised representation of the emissions applicable to further analysis. To meet these objectives a screening program was conducted in which slurry materials were collected and, within 24 h, incubated at near-ambient temperature to determine CH_4_ production rates. The slurry materials were further characterised with respect to VS composition as a basis for model parameterisation, and model predictions were compared with current CH_4_ emission estimates for these manure categories based on the IPCC methodology.

## Materials and Methods

Liquid manure (slurry) from dairy and pig farms delivering slurry to a centralised biogas facility in Thorsø, Western Denmark was collected for this study. Based on statistical information about livestock production and housing types in Denmark [15], representative pig and cattle farms were contacted, followed by on-site interviews and inspection. From this survey seven farms were selected for the monitoring program and farmers contacted to gain permission for sampling. Table 1 shows animal category, housing, and slurry system, as well as slurry collection frequency, for each farm. Also shown are the number of visits and individual samples collected per visit, which amounted to a total of 12 cattle slurry and 27 pig slurry samples; more samples could be obtained on pig farms which had several production lines in separate sections.

**Table 1:** Overview of farms visited; all farms supplied slurry to Thorsø Biogas Plant.

Farm ID ^1^^)^	Animal category	Housing system	Slurry system	Collections per wk	No. visits	Samples per visit
G1	Dairy cattle	Cubicles	Ring channel	3	2	1
G2	Dairy cattle	Cubicles	Ring channel	1	2	1
G5	Dairy cattle	Cubicles	Scrapers + backflush	3	2	2
G6	Dairy cattle	Cubicles	Scrapers + backflush	2	2	2
G3	Finishing pigs	Partly slatted	Pull-plug	1	1	1
G4	Finishing pigs	Partly slatted	Pull-plug	2	2	6
G7	Farrowing sows	Indiv confinement	Pull-plug	2	2	6
G7	Farrowing sows	Loose, indiv confinement	Pull-plug	2–3	1	5
G3	Piglets	Partly slatted	Pull-plug	1	1	1

^1)^ Geographical coordinates: G1–56°21'10'' N, 9°48'10'' E; G2–56°18'02'' N, 9°43'42'' E; G3–56°20'13'' N, 9°53'13'' E; G4–56°21'06'' N, 9°45'26'' E; G5–56°21'29'' N, 9°52'12'' E; G6–56°16'11'' N, 9°45'24'' E; G7–56°21'02'' N, 9°49'06'' E.

Slurry pits on pig farms were in most cases 40–60 cm deep and less than half full at sampling. In contrast, on dairy farms G1 and G2 the ring channel was always kept nearly full since a large liquid phase is needed for mobilisation and transport of manure organic matter when exported. On dairy farms G5 and G6 the pit was backflushed with slurry from a pre-tank. The number of samples which could be processed within this study was too limited to characterise individual production systems, and samples were therefore categorised as either cattle slurry or pig slurry.

### Sampling procedure

Sampling took place on several days between 18 November and 11 December 2014 to allow processing of all samples within 24 h. Separate manual bilge pumps were used on each farm to collect 3-liter samples in 5-liter buckets from below slatted floors, pooling several subsamples from different positions to give the final sample. Air and slurry temperatures were registered at or close to the positions where slurry was sampled using a SAF-T-LOG^®^ HACCP ThermoMeter with a 1.4-meter probe and an accuracy of 0.4°C (ThermoWorks, Lindon, UT). Upon return to the laboratory slurry samples were stored outside until the following day (mean night temperatures between 0 and 7°C).

### Determination of CH_4_ production rates

Slurry processing for determination of CH_4_ production rates largely followed the procedure described by Elsgaard et al. [16]. Three-gram portions of each slurry material were transferred to eight 28 mL test tubes while flushing the headspace with N_2_ following the Hungate approach [17]. Each test tube (subsample) was immediately closed with butyl rubber septum and crimp seal and placed on ice to temporarily arrest methanogenesis while completing sample preparation. Two subsamples were shaken vigorously for 1 min to release dissolved CH_4_ and then sampled as described below for gas chromatographic analysis; this information was later used as background when calculating rates of CH_4_ production during incubation. The other six subsamples were incubated at a temperature close to the temperatures recorded in slurry pits at the respective farm units.

The first batch of samples was incubated in a thermo-gradient incubator [18]; due to technical problems subsequent incubations were done using water baths operated at temperatures of approximately 10 and 20°C, respectively. The exact average temperature of water baths was certified with data from immersed temperature loggers (Hobo Pendant Temperature Data logger; Onset Computer Corp., Bourne, MA). An incubation time of 17 h was used based on the results of Elsgaard et al. [16].

Around 0.5 h before end of incubation up to 3 mL N_2_ was added to the headspace to ensure sufficient gas volume for sampling. By the end of incubation samples were shaken to release dissolved gases, and then a 10 mL glass syringe was used to determine headspace volume at atmospheric pressure. Then a 3 mL sample of the headspace gas was transferred to a 6 mL Exetainer (Labco Inc., Lampeter, UK) pre-equilibrated to atmospheric pressure with N_2_ (i.e., the vials were pressurised for analysis). Methane concentrations were determined on an Agilent 7890 gas chromatograph (GC) with CTC CombiPal autosampler (Agilent, Nærum, Denmark). For CH_4_ analysis the GC had a 2 m backflushed pre-column with Hayesep P connected to a 2 m main column with Poropak Q. The main column was connected to a flame ionization detector (FID). The carrier gas was N_2_ at a flow rate of 45 mL min^−1^. The FID was supplied with 45 mL min^−1^ H_2_, 450 mL min^−1^ air and 20 mL min^−1^ N_2_. Temperatures of injection port, columns and FID were 80, 80 and 200°C, respectively. The method detection limit for CH_4_ was 0.2 μL L^-1^. Observed concentrations ranged from 20 to 7000 μL L^-1^; the detector response was linear over this range (r^2^ = 0.999) as determined by standards prepared from a reference gas with 47,500 μL L^-1^ CH_4_ (AGA; Copenhagen, Denmark).

The measured CH_4_ production rates were corrected to the exact temperature of slurry pit or ring channel at the time of sampling using the equation:

$$ln\left(\frac{k_{2}}{k_{1}}\right)=-\left(\frac{E_{a}}{R}\right)\left(\frac{1}{T_{2}}-\frac{1}{T_{1}}\right)$$ (1)

where *k*_*1*_ and *k*_*2*_ are the measured and corrected CH_4_ production rate (mg CH_4_ kg^-1^ VS h^-1^), respectively, *E*_*a*_ is activation energy (81 J mol^-1^ [16]), *R* is the universal gas constant (8.314 J K^-1^ mol^-1^), and *T*_*1*_ and *T*_*2*_ are the temperatures (K) during laboratory incubation and in the slurry pit, respectively.

### Slurry analyses

Slurry pH and electrical conductivity were measured using a pH/conductivity meter (CyberScan PC 300, EUTECH Instruments, Landsmeer, Netherlands). Slurry dry matter was determined by drying of approx. 10 g slurry fresh wt. subsamples at 105°C for 24 h. Slurry VS was determined by incineration of the dried material at 450°C for 5 h.

An estimate of degradable VS (VS_d_) in the slurry materials was needed for model calculations (see below). Samples collected in slurry pits are characterised by an unknown degree of degradation, and therefore data from the literature representing fresh excreta, such as those derived by Sommer et al. [14], could not be used. Instead an aerobic assay was adopted in which VS_d_ was estimated from the ratio between short-term CO_2_-C production during aerobic incubation and total organic carbon (TOC) in each slurry sample. Total organic carbon (g C kg^-1^ fresh wt.) was analysed in triplicate by a cuvette test LCK 387 (DR 3900, HACH Lange; Düsseldorf, Germany) using a standard method (EN 1484). For determination of VS_d_, 5 g slurry samples were surface-applied to sandy loam soil packed in 100-cm^3^ cylinders to a bulk density of 1.2 g cm^-3^, and with soil moisture adjusted to 40% water-filled pore space. Preliminary tests showed that aerobic degradation of slurry VS would predominate under these assay conditions. The samples were incubated in a Respicond VI respirometer (Nordgren Innovation AB, Bygdeå, Sweden) at 20°C for 14 d where potential CO_2_ evolution was determined by hourly measurements of conductivity in alkaline traps. The CO_2_ accumulated over time (*Y*_*t*_, g C kg^-1^ fresh wt. slurry), corrected for background emissions from the soil, was used to estimate the asymptotic maximum (*Y*_*max*_) with an exponential model:

$$Y_{t}=Y_{max}\;(1-e^{-kt})+y_{0}$$ (2)

where *k* is a rate constant (h^-1^), t is time (h), and y_0_ represents the offset on the y axis which may include degassing of carbonates dissolved in the slurry. *Y_max_* (g C kg^-1^ fresh wt. slurry) was determined by curve fitting in SigmaPlot 11.0 (Systat Software Inc.), but disregarding the 0–12 h period. Then VS_d_ (kg) was calculated from total VS (kg) and the ratio between maximum CO_2_-C evolved and TOC in the slurry:

$${VS}_{d}=\frac{Ymax}{TOC}\;VS$$ (3)

### Modeling of CH_4_ production rates

For comparison with experimental results, CH_4_ production rates were calculated using the algorithm proposed by Sommer et al. [14]:

$$F_{t}=({VS}_{d}+0.01{VS}_{nd})\;e^{\left(lnA-\frac{E_{a}}{RT}\right)},$$ (4)

where *F*_*t*_ is CH_4_ production rate (mg CH_4_ kg^-1^ VS h^-1^), VS_nd_ (kg) is the remaining fraction of total VS which is virtually nondegradable during in-house storage, and *E*_a_ and lnA (mg kg^-1^ VS h^-1^) are Arrhenius parameters (units of *E*_a_, *R* and *T* as in Eq 1). The model thus assumes that degradation of VS_nd_ is 100-fold slower than the degradation of VS_d_. The model parameters (VS_d_, *E*_a_ and lnA) used by Sommer et al. [14] to describe storage of fresh excreta are shown in Table 2, together with parameter estimates for slurry in pits derived from this study.

**Table 2:** Key parameters for in-house manure storage of the model proposed by Sommer et al. [14] to describe storage of fresh excreta, and experimentally derived parameters for stored slurry based on this study and Elsgaard et al. [16].

	Slurry type	Sommer et al. (2004)	This study
**VS**_**d**_ **(kg kg**^**-1**^ **VS)**	pig	0.89	0.51a (0.45–0.57)^§^
	cattle	0.46	0.33b (0.29–0.36)
***E***_**a**_ **(kJ mol**^**-1**^**)**	pig	112.7	81.0 (74.9–87.1)^#^
	cattle	112.7	81.0 (74.9–87.1)^#^
**lnA (g CH**_**4**_ **kg**^**-1**^ **VS h**^**-1**^**)**	pig	44.22	31.3a (31.0–31.7)
	cattle	44.29	31.2a (30.7–31.8)

^§^ Numbers in parentheses are 95% confidence limits.

^#^ From Elsgaard et al. (2016).

Methane production rates were also calculated using a new parameterisation based on experimental data. An activation energy (*E*_a_) of 81 kJ mol^-1^ was adopted from Elsgaard et al. [16] who found no significant difference in the temperature response of a cattle slurry, a pig slurry, fresh digestate and stored digestate over the temperature range 5–35°C. The frequency factor of the Arrhenius equation, A, is related to substrate quality and methanogenic potential and therefore unique to each material. Individual estimates of lnA for each slurry material were calculated by rearranging Eq 4:

$$lnA=ln\left[\frac{F_{t}}{\left({VS}_{d}+0.01{VS}_{nd}\right)}\right]+\frac{E_{a}}{RT},$$ (5)

hence estimates of lnA were not related to total VS, but only to the fraction of VS (i.e., VS_d_ + 0.01VS_nd_) that was considered to be the substrate for methanogenesis.

### Statistical analyses

Individual characteristics of pig and cattle slurry, including CH_4_ production rate, were compared by *t* tests following transformation when required to achieve normal distribution and homogeneity of variance [19]. Model sensitivity to parameter estimates derived from experimental results was evaluated using a sensitivity ratio (SR), which calculates the relative change in model output, i.e., predicted CH_4_ production rate, for a given deviation in parameter estimate [20]. Here, parameter ranges of lnA, VS_d_ and slurry temperature corresponding to 95% confidence limits were used:

$$SR=\left(\frac{y_{+95\%\;C.L.}-y_{-95\%\;C.L.}}{x_{+95\%\;C.L.}-x_{-95\%\;C.L.}}\right)∙\left(\frac{x_{obs}}{y_{obs}}\right),$$ (6)

where *y* is CH_4_ production rate in cattle or pig slurry, and *x* the parameter evaluated.

## Results and Discussion

The housing systems visited in this study (Table 1) represented 52% of all LU in Denmark where 78% of livestock manure is handled in liquid form. Ring channels and passively drained slurry pits are only partly emptied when slurry is exported, and therefore adapted methanogens will be present to inoculate fresh excreta [4]. This is important because then physical and chemical characteristics of the slurry will mainly determine the CH_4_ production potential.

Summary statistics for selected slurry properties are presented in Table 3; details about individual samples are included as S1 Table. Slurry temperature in pig houses ranged from 14.8 to 22.3°C, and in cattle houses from 5.5 to 12.3°C (with one outlier at 16°C which was probably caused by inflow of water used for cleaning of an adjacent milking parlour). Cattle slurry was characterised by higher dry matter and volatile solids content compared to pig slurry. Total organic C, determined by a wet destruction method, was well correlated with slurry VS (S1 Fig) (r = 0.91, n = 38), and there was on average 0.42 and 0.44 kg C kg^-1^ VS in cattle and pig slurry, respectively. According to Derikx et al. [21] there is a potential for loss of volatile fatty acids during oven drying which could lead to underestimation of VS. Since TOC is unaffected by this source of error, loss of volatile fatty acids would result in TOC:VS ratios being overestimated. The average carbon contents of crude lipid, crude protein and carbohydrates are around 77, 53 and 40–44%, respectively [22]. These values are higher than or similar to the TOC:VS ratios observed here, suggesting that losses of VS during oven drying were not a major source of error.

**Table 3:** Selected slurry characteristics, means with 95% confidence limits. Different letters within a row indicate that differences were significant (P<0.05). Values for individual slurry samples are shown in S2 Table.

	Unit	Cattle slurry	Pig slurry
**Dry matter**	g kg^-1^ fresh wt.	91a (63–119)^§^	58b (42–75)
**Volatile solids, VS**	g kg^-1^ fresh wt.	65a(51–78)	37b (26–48)
**Degradable VS, VS**_**d**_	kg kg^-1^ VS	0.33a (0.29–0.36)	0.51b (0.45–0.57)
**Total organic C**	g kg^-1^ fresh wt.	27a (20–34)	15b (11–19)
**Conductivity**	mS cm^-1^	9.7a (7.8–11.6)	32.3b (21.0–43.5)
**pH**		7.2a (6.9–7.5)	7.3a (7.1–7.5)
**Slurry temperature**	°C	9.8a (8.2–11.4)	18.6b (17.8–19.4)

^§^ Numbers in parentheses are 95% confidence limits.

### Methane production rates

In eight of the 39 slurry samples collected for this study the DM content was less than 50%, or more than 200%, of the DM recorded in batches of slurry delivered to Thorsø Biogas Plant during the previous year (S3 Table). These eight samples were deemed unrepresentative and excluded from the estimation of CH_4_ emissions from pits with pig and cattle slurry. Methane production rates of the remaining 31 samples (Table 4) were corrected to ambient temperature using Eq 1 (Materials and Methods). The difference between temperatures of slurry pits and incubations ranged between -3.9 and +3.3°C; the maximum relative correction of observed CH_4_ production rates was 35%.

**Table 4:** Methane production rates (MPR) corresponding to the temperature in slurry channels at the time of collection. Data shown are mean and 95% confidence limits (C.L.) of six replicates, and coefficients of variation (C.V.).

Sample ID	Slurry type	Ambient slurry temperature	MPR (mg CH_4_ kg^-1^ VS h^-1^)	C.V. (%)
2	Pig	16.9 (-1.1) ^#^	12.7 (9.1–16.2)^§^	14
3	Pig	18.4 (-0.3)	44.3 (38.2–50.3)	7
5	Pig	14.8 (0.4)	25.4 (15.2–35.5)	21
9	Pig	18.4 (1.2)	116.4 (106.6–126.2)	4
10	Pig	17.5 (0.3)	90.5 (76.1–104.8)	8
11	Pig	19.4 (-0.6)	115 (105.2–124.8)	4
12	Pig	20.1 (0.1)	130.4 (92.3–168.4)	15
13	Pig	22.3 (2.3)	231.9 (213.6–250.1)	4
14	Pig	20.2 (0.2)	92 (62.2–121.7)	17
15	Pig	17.7 (-2.3)	39.3 (14.4–64.1)	32
16	Pig	21 (1)	70.8 (46.6–94.9)	17
17	Cattle	5.5 (-3.5)	3.7 (2.9–4.4)	12
18	Cattle	9.1 (0.1)	13 (4.1–21.8)	35
19	Cattle	9.4 (0.4)	14.3 (11.1–17.4)	11
20	Cattle	10.5 (1.5)	39 (32.5–45.4)	8
21	Cattle	9.3 (0.3)	12.2 (5.3–19)	28
23	Pig	20.6 (-1.4)	65.6 (25.6–105.5)	31
24	Pig	21.4 (-0.6)	79.1 (67.7–90.4)	7
25	Pig	22 (0)	147.6 (116.4–178.7)	11
26	Pig	20 (-2)	99.3 (91.8–106.7)	4
27	Pig	18.1 (-3.9)	28.9 (10.4–47.3)	32
28	Pig	18.6 (-3.4)	83 (71.2–94.7)	7
29	Cattle	7.4 (-1.6)	6.5 (-3.4–16.4)	24
30	Cattle	8.8 (-0.2)	7.1 (0.8–13.3)	11
31	Cattle	9.7 (0.7)	17.1 (14.7–19.4)	7
32	Cattle	9 (0)	29.3 (26.5–32)	5
33	Cattle	10.7 (1.7)	2.3 (0.7–3.8)	32
34	Cattle	20.6 (-1.4)	28 (24.8–31.1)	6
36	Pig	18.9 (1.9)	57 (54.8–59.1)	2
37	Pig	19.4 (2.4)	84.8 (68.1–101.4)	10
38	Pig	16.4 (-0.6)	25.3 (18.8–31.7)	13

^#^ Deviations from ambient during assay are shown in parentheses.

^§^ Numbers in parentheses are 95% confidence limits.

**Fig 1:**
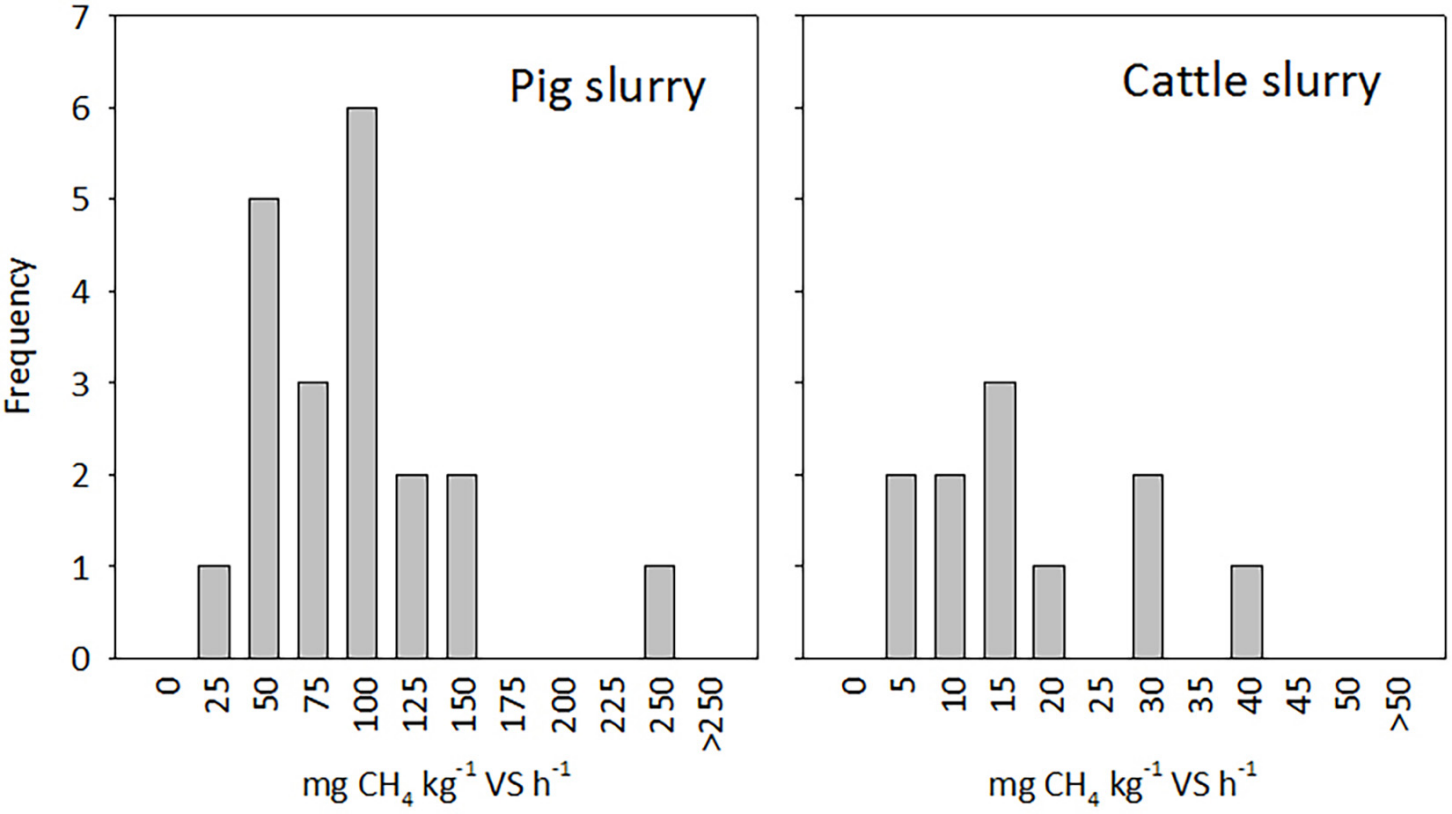
Frequency distribution for observed methane production rates in pig and cattle slurries. Values on x-axis represent lower boundaries of bins.

Histograms of the rate distributions for pig and cattle slurry are shown in Fig 1. Shapiro-Wilks tests for normality were accepted for both slurry types. Mean CH_4_ production rates observed with cattle slurry were around five times lower than rates observed with pig slurry. This was partly explained by the lower storage temperature in cattle houses with passive ventilation, but degradability of VS in cattle excreta was probably also lower, as indicated by VS_d_ (Table 3). For pig slurry a negative relationship between time since the last emptying and CH_4_ production rate was indicated, but it was not significant (*p =* 0.27, *n* = 18).

The laboratory-based CH_4_ production rates observed in this study may be compared with rates derived from the national inventory of agricultural GHG emissions [23]. Here daily excretion of VS by pigs and cattle, and the emission of CH_4_ from manure management per head per year, is reported (Table 5), and based on this information CH_4_ emissions per kg VS of 0.032 and 0.015 kg CH_4_ kg^-1^ VS from pig and cattle slurry, respectively, can be estimated. In the present study daily CH_4_ emission rates per kg VS were determined. Assuming a retention time in slurry pits of 15 and 30 d for pig and cattle slurry, respectively [14], total emissions from slurry pits of 0.030 and 0.011 kg CH_4_ kg^-1^ VS were estimated which are only sligthly lower than the estimates of total CH_4_ emissions from manure management derived from the national inventory. This agreement suggests that the estimates of CH_4_ emissions from manure management in the national inventory (including emissions from both confinements and storage facilities) are at a realistic level. The fact that CH_4_ emissions from slurry pits observed here were only slightly lower than total estimated emissions from manure management is evidence that the proportion of CH_4_ emitted from slurry pits is substantial, and that possibly total CH_4_ emissions from pits and outside storage tanks are currently underestimated.

**Table 5:** National inventory *vs*. observations. Methane emissions from manure management, based on the national inventory of GHG emissions from Danish agriculture [23], were compared with observed estimates of CH_4_ emissions from slurry pits.

	Pig slurry	Cattle slurry	Unit
*Manure management*, *DK inventory*:			
**Daily VS excretion**	0.2	6.2	kg VS hd^-1^ d^-1^
**Methane emissions, annual**	2.3	34	kg CH_4_ hd^-1^ yr^-1^
**Methane emission per kg VS**	0.032	0.015	kg CH_4_ kg^-1^ VS
*Slurry pits*, *this study*:			
**Methane emissions, daily**	1.97 (1.39–2.54) ^§^	0.38 (0.19–0.57)	g CH_4_ kg^-1^ VS d^-1^
**Retention time**	15	30	d
**Methane emission per kg VS**	0.030	0.011	kg CH_4_ kg^-1^ VS

^§^ Numbers in parentheses are 95% confidence limits.

### Model parameters: Degradable VS (VS_d_)

Sommer et al. [14] estimated the degradability of VS in fresh excreta at 0.89 and 0.46 kg VS_d_ kg^-1^ VS in pig and cattle slurry, respectively. These values can be related to the default values for *B*_*0*_ (i.e., the maximum CH_4_ producing capacity of VS excreted; *B*_*0*_ is also referred to as biochemical methane potential, BMP) proposed in the IPCC methodology ([5]; Annex 10A.2) which are 0.45 m^3^ CH_4_ kg^-1^ VS for pigs, and 0.24 m^3^ CH_4_ kg^-1^ VS for dairy cattle: At normal temperature and pressure (NTP), 1 m^3^ CH_4_ gas is equivalent to 0.503 kg CH_4_-C, and assuming a 60:40 molar ratio between produced CH_4_ and CO_2_ during *B*_*0*_ measurement [24], this would correspond to a loss of 0.838 kg C m^-3^ CH_4_. Using the observed proportions of C in VS, i.e., 0.44 kg C kg^-1^ in pig slurry and 0.42 kg C kg^-1^ VS in cattle slurry, the *B*_*0*_ of 0.45 m^3^ CH_4_ kg^-1^ VS for pigs corresponds to a VS degradability of 0.86 kg kg^-1^ VS, and the *B*_*0*_ of 0.24 m^3^ CH_4_ kg^-1^ VS for cattle corresponds to a VS degradability of 0.48 kg kg^-1^ VS. Thus, pools of VS_d_ in pig and cattle slurry defined by Sommer et al. [14] are largely equivalent to *B*_*0*_ values as defined in the IPCC methodology.

The slurry collected in pits was partly degraded at the time of collection, and therefore characteristics of fresh excreta did not apply. Instead an estimate of VS_d_ was obtained using a 14-day biodegradation assay to monitor CO_2_-C evolution; results and curve fits are shown in the online Annex (S2 Fig, S3 Fig). Maximum CO_2_-C (*y*_*max*_ in Eq 2, Materials and Methods) was related to TOC as an estimate of VS_d_ in individual slurry samples (Eq 3, Materials and Methods). The aerobic assay was preferred partly because of the shorter time required compared to anaerobic batch incubation for determination of *B*_*0*_, and partly because of the complications associated with determination of *B*_*0*_ [25–27]. It is well accepted that both aerobic degradation and anaerobic degradation of organic matter follow first-order degradation kinetics, but with different specific reaction constants [28]. Lesteur et al. [26] reviewed several methods for estimating anaerobic biodegradability of an organic substrate and concluded that VS degradation during a 5 d aerobic incubation as determined by oxygen (O_2_) uptake and/or CO_2_ evolution was in good agreement with a 21 d anaerobic incubation. Ponsa et al. [29] also found a strong correlation between aerobic and anaerobic stability indices for municipal solid waste materials. On the other hand, the current manure materials differed widely in DM content and soil infiltration after surface application in the VS_d_ assay, and further method development is probably needed to optimise the determination of VS_d_.

The results of VS_d_ averaged 0.51 kg VS_d_ kg^-1^ VS for pig slurry and 0.33 kg VS_d_ kg^-1^ VS for cattle slurry (Table 3); this was, respectively, 43 and 28% lower than the VS_d_ concentrations in fresh excreta as defined by Sommer et al. [14] (cf. Table 2). These results suggested that significant VS degradation had already occurred during the short-term storage in slurry pits.

### Model parameters: Arrhenius parameters (E_a_ and lnA)

An *E*_*a*_ value of 81 kJ mol^-1^ was adopted from Elsgaard et al. [16] which was much lower than the value of 112.7 kJ mol^-1^ derived by Sommer et al. [14] from three different slurry storage experiments. The storage experiments used by Sommer et al. [14] ranged in duration from a few days and up to one year, and hence factors other than temperature could have influenced the relationship. For example, Petersen et al. [30] calculated tentative *E*_a_ values of only 21–25 kJ mol^-1^ for CH_4_ emissions across winter and summer storage experiments with pig slurry, and it was concluded that depletion of degradable VS during summer storage probably resulted in underestimation of the response to higher temperatures. Other models of CH_4_ emissions from manure, e.g. [31,32], have used a value of 63 kJ mol^-1^ derived from a number of different studies covering a temperature range from 15 to 60°C. In contrast, the *E*_a_ value reported by Elsgaard et al. [16] was derived from complete temperature response profiles of individual manure materials and digestates, and representing a temperature range (5 to 35°C) relevant for manure storage, which indicates that this is currently the most robust estimate available for the temperature response of CH_4_ production in livestock slurry during storage.

The lnA values used by Sommer et al. [14] were also very different from those determined experimentally in this study. It should, however, be noted that Sommer et al. [14], due to limited availability of data for parameterization, fitted lnA to produce the same annual emission as the IPCC methodology. The parameter lnA reflects a potential for CH_4_ production that is influenced by chemical and biological characteristics of the manure material (livestock category, age, and adaptation of the methanogenic microbial community). In the present study lnA was derived for each slurry sample using Eq 5 (cf. Materials and Methods), and the observed lnA averaged 31.3 and 31.2 g CH_4_ kg^-1^ VS h^-1^ for pig and cattle slurry, respectively (Table 2). In comparison, the lnA values observed by Elsgaard et al. [16] in pig slurry and beef cattle slurry after storage for several months were 31.1 and 33.3 g CH_4_ kg^-1^ VS d^-1^, corresponding to 27.9 and 30.1 g CH_4_ kg^-1^ VS h^-1^. The pig and cattle slurry analysed by Elsgaard et al. [16] had been collected during at least six months, and so the lower values of lnA compared to samples from slurry pits probably reflected the more advanced stage of decomposition.

### Parameter sensitivity

**Fig 2:**
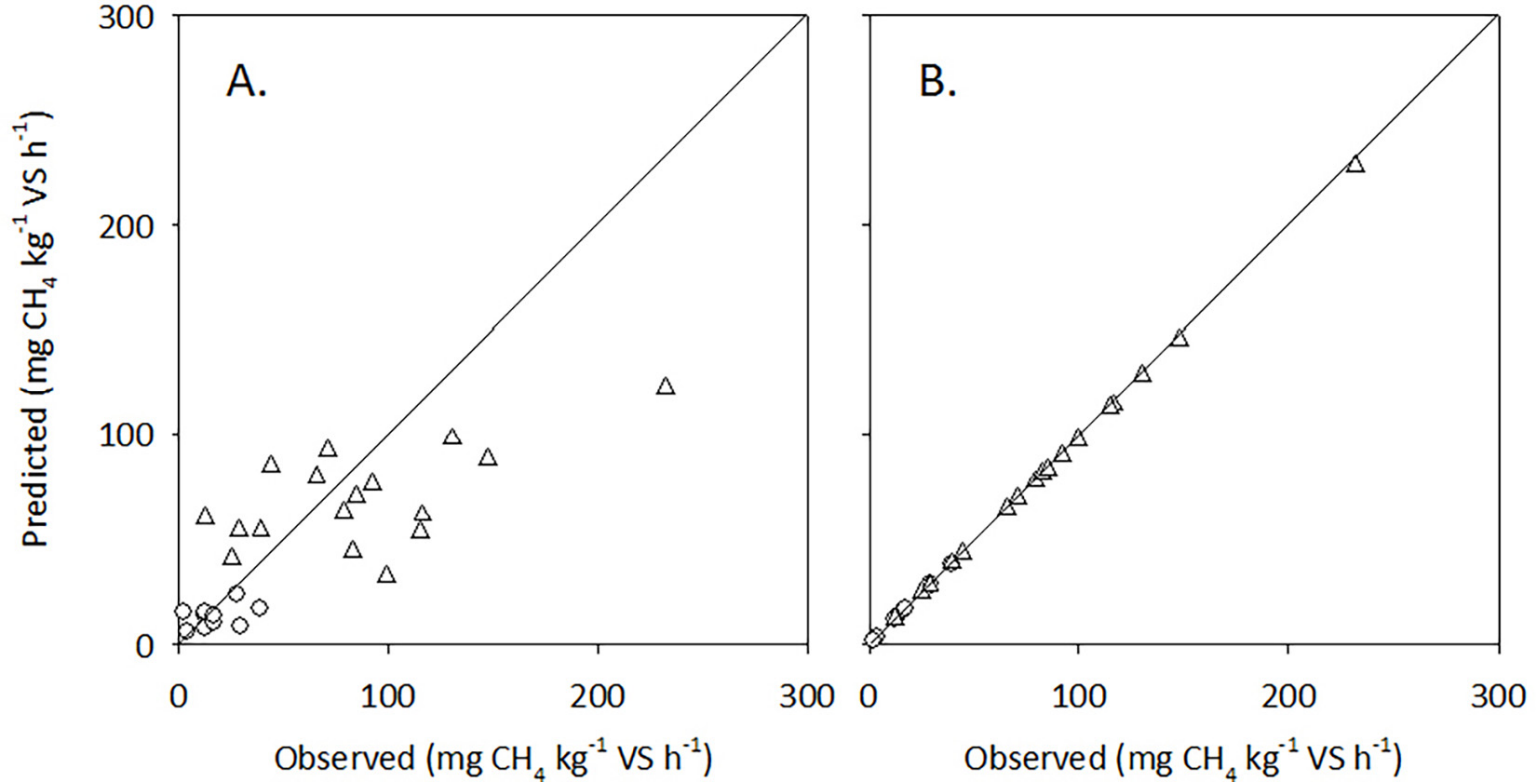
Observed *vs*. calculated CH_4_ production rates (g CH_4_ kg^-1^ VS d^-1^) in slurry from pits under confined pigs (triangles) and cattle (circles). In panel A the calculated rates were based on average lnA and individually determined VS_d_, whereas in panel B individual values for lnA and average VS_d_ were used.

The response of predicted CH_4_ production rates to uncertainties in parameter estimates were evaluated by calculation of sensitivity ratios (Eq 6, Materials and Methods) for each of the parameters lnA, VS_d_ and slurry temperature (Table 6). In each case the 95% confidence limits were selected as upper and lower boundaries of parameter uncertainty. Predicted CH_4_ production rates were only moderately sensitive to uncertainties in the estimation of VS_d_ or slurry temperature, but dramatically affected by uncertainty in the estimation of lnA. This confirms that lnA must be determined experimentally for slurry representing a given livestock category and production system, whereas the precision required is smaller for VS_d_. This is illustrated in Fig 2 where the observed lnA or VS_d_ for individual slurry samples were substituted by the average observed value for lnA (Fig 2A) or VS_d_ (Fig 2B). Clearly, deviations between predicted and observed values were large when using average values of lnA for pig and cattle slurry, whereas the adoption of average VS_d_ rather than individual values had little effect on the prediction. This suggests that adoption of a typical VS_d_ estimate for a given livestock category and production system may be acceptable if combined with observed CH_4_ production rates, which allows calculation of lnA for individual slurry samples. This further implies that, with a representative set of slurry samples from a given livestock category and production system, it is possible to derive a robust estimate of mean lnA with confidence limits which can be used in scenario analyses.

**Table 6:** Sensitivity ratios calculated for model parameters lnA, VS_d_ and slurry temperature.

	lnA	VS_d_	Temperature
**Pig slurry**	33.45	0.98	2.26
**Cattle slurry**	41.06	1.11	2.31

### Implications for the estimation of CH_4_ emission and VS loss

The current IPCC methodology for estimating CH_4_ emissions from slurry pits under animal confinements relies on very basic emission factors. On-farm verification of this source is extremely difficult, and an estimation method linking CH_4_ production rates to quantifiable slurry properties would be an attractive alternative (Fig 1). The approach adopted in this study, corresponding to a Tier 3 method in the IPCC terminology, may represent such an alternative for estimating current CH_4_ emissions from slurry below animal confinements. Furthermore, the method could help compare the mitigation potential of contrasting management practices, and effects of manure treatment technologies such as anaerobic digestion.

Observed CH_4_ production rates were well described using the empirical model with experimentally derived parameters for VS_d_, slurry temperature in the pit, an independent estimate of *E*_a_ [16], and lnA values calculated for individual slurry samples (Fig 2B). This is not a prediction model for estimating CH_4_ emissions from a wider range of slurry materials, but rather a generalisation of observed CH_4_ production rates that will represent a given livestock production system, as defined by livestock category, feeding, housing design, and manure management. The information required for individual slurry samples include CH_4_ production rate, temperature of slurry in the pit at sampling, and total and degradable VS. Our results indicate that from this information a robust estimate of CH_4_ emissions from a given source may be obtained. The number of slurry samples needed in each case will depend on the diversity of production systems and variability of slurry characteristics.

It was stated above that precise estimation of VS_d_ is less critical for the parameterisation of Eq 4 to characterise CH_4_ emissions from slurry pits for a given livestock category. However, if model parameters are to be used for scenario analyses, the loss of VS and VS_d_ over time is still important, since prediction of CH_4_ emissions on a daily basis also requires an estimate of daily VS loss. Carbon in VS is lost as both CH_4_ and CO_2_, and the proportions of CH_4_ and CO_2_ can vary depending on storage conditions. For example, significant aerobic degradation of carbon in VS to CO_2_ may take place near the slurry-air interface, as demonstrated by Møller et al. [33]. Patni and Jui [34] also concluded that aerobic degradation was responsible for declining VS concentrations in the top layer during storage of cattle slurry. Significant degradation of carbon in VS to CO_2_ may also be inferred from numerous slurry storage experiments reporting CH_4_:CO_2_ ratios of 0.1–0.3, e.g. [35–38]. Factors such as design of slurry pits, ventilation and retention time will all influence the exposure of slurry to atmospheric oxygen and, presumably, the balance between CO_2_-C and CH_4_-C emitted from slurry pits.

There is further evidence that the CH_4_:CO_2_ ratio of gases emitted from stored slurry declines with decreasing temperature [33,35]. This would be consistent with a recent report that temperature sensitivity of methanogenic communities is higher than that of ecosystem respiration, i.e., 0.93 and 0.65 eV, respectively, corresponding to *E*_a_ values of 89 and 63 kJ mol^-1^, which implies that CH_4_:CO_2_ ratios will decline with temperature [39].

An attempt was made to assess proportions of CH_4_ and CO_2_ emitted assuming VS_d_ of 0.89 and 0.46 kg kg^-1^ VS in fresh excreta from pigs and cattle, and retention times in the pit of 15 and 30 d for pig and cattle slurry, respectively [14]. Using the observed mean slurry temperatures in pig and cattle slurry with 95% confidence limits (Table 3), and the revised Arrhenius parameters from this study (Table 2), the daily loss of total VS and VS_d_ was calculated for different proportions of CH_4_ and CO_2_. Specifically, CH_4_/(CH_4_ + CO_2_) ratios of 0.05, 0.1, 0.3 and 0.6 were used, corresponding to 5, 10, 30 or 60% of carbon in VS being emitted as CH_4_, and the rest as CO_2_.

**Fig 3:**
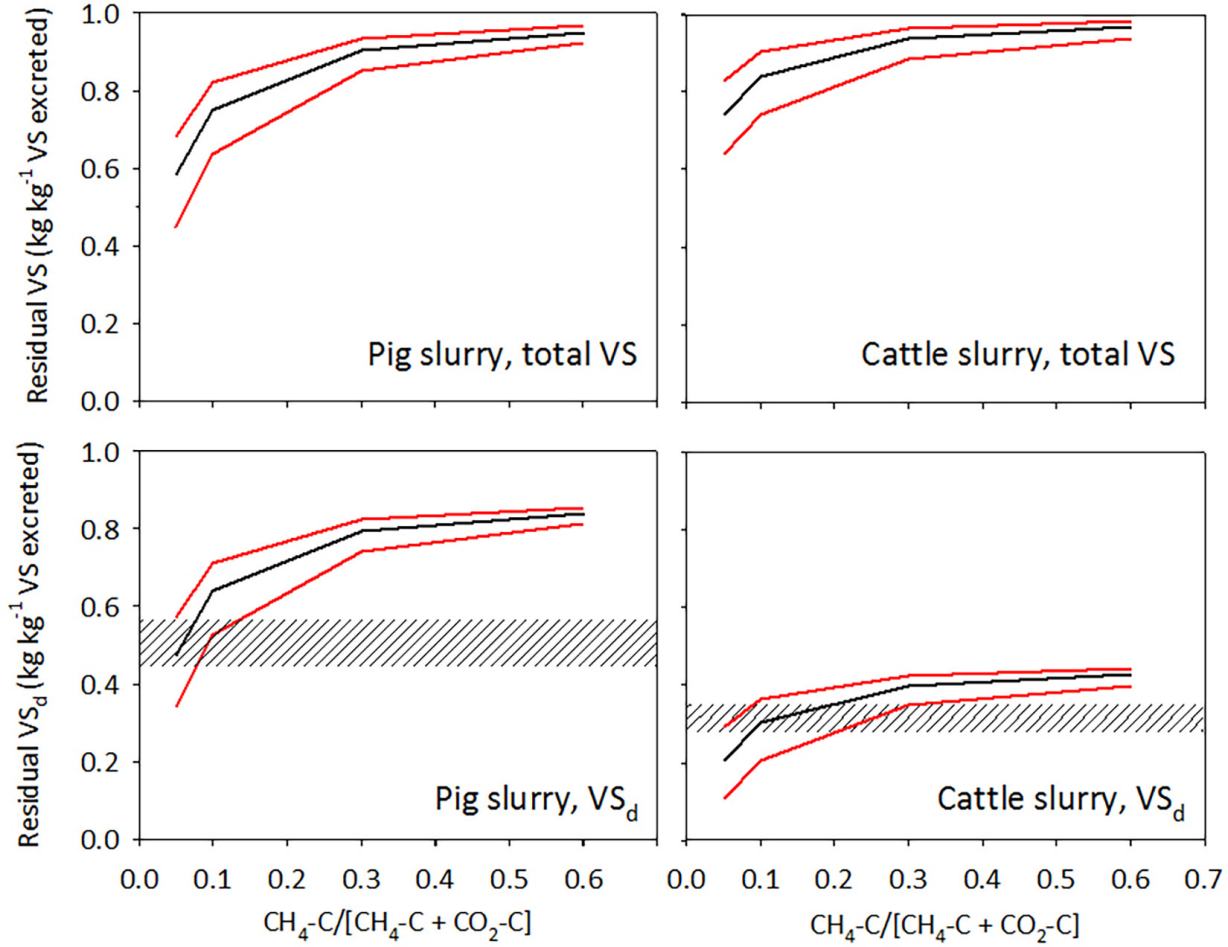
Using Eq 4, and with parameters for pig and cattle slurry determined in this study, the loss of total VS and VS_d_ were estimated for different assumptions regarding the proportions of CH_4_ and CO_2_ derived from VS degradation. The proportion of CH_4_-C in total C lost (CH_4_-C + CO_2_-C) was set to 5, 10, 30 or 60%. Loss of VS was then estimated as: $loss\;(kg\;d^{-1})=\frac{{CH}_{4}\;(kg\;C\;d^{-1})}{\frac{\left({CH}_{4}-C\right)}{{CH}_{4}-C+{CO}_{2}-C}\times \frac{TOC}{VS}}$. The calculation was done separately for VS_d_ and VS_nd_ with daily time steps. In each plot the black line represents estimated average VS or VS_d_ remaining at sampling at the observed mean temperature in slurry samples, and the red lines the estimates corresponding to 95% confidence limits of slurry temperature. The gray shaded areas in lower plots represent the 95% confidence range of observed VS_d_ in pig and cattle slurry, respectively. The calculated residual VS_d_ and VS_nd_, with daily time steps, are shown in S4 Table.

Total residual VS calculated for each CH_4_/(CH_4_ + CO_2_) ratio are shown in the top panel of Fig 3, and in the bottom panel residual concentrations of VS_d_. Here, the gray hatched area represents the 95% confidence range of the experimentally determined residual VS_d_, while the black and red lines represent, respectively, the average and 95% confidence limits of modeled residual VS_d_. The overlapping confidence ranges then indicate the range of CH_4_/(CH_4_ + CO_2_) ratios consistent with the observed residual VS_d_ concentrations. According to these plots 5–15% of carbon from pig slurry VS was emitted as CH_4_, and 85–95% as CO_2_. For cattle slurry 5–35% of carbon in VS degraded was emitted as CH_4_, and 65–95% as CO_2_. These ranges seem to confirm the results from storage experiments cited above, and it must be concluded that loss of VS as CO_2_ are substantial and must be taken into account when estimating the loss of degradable VS in slurry pits. In this example, VS and VS_d_ in fresh excreta were not determined experimentally, and hence more work is needed to verify proportions of CH_4_ and CO_2_ in VS degradation products, as influenced by livestock category and storage conditions.

## Conclusions

This study presented a new *in-vitro* method for quantifying CH_4_ production rates in slurry stored in pits on livestock farms. Observed CH_4_ production rates compared well with those currently implied in the national inventory, suggesting that CH_4_ emissions from slurry pits can be estimated from slurry samples collected and analysed within 24 h at ambient temperature. An empirical model can be parameterised for individual livestock production systems based on CH_4_ production rates and readily measured slurry characteristics, thus providing a generalised form applicable to estimation of methane conversion factors and scenario analysis. The need to account for VS degradation to both CH_4_ and CO_2_ was emphasised, since this has important implications for both CH_4_ emission estimates, and for the estimation of biogas potentials of livestock slurry.

## Supporting Information

S1 Fig Relationship between volatile solids in cattle (circles) and pig (triangles) slurry materials and TOC. (PDF)

S2 Fig Evolution of CO_2_-C from cattle slurry samples during aerobic decomposition. The blue lines represent observations, corrected for background emissions from the soil, while the red lines represent 95% confidence limits of model fits.
(PDF)

S3 Fig Evolution of CO_2_-C from pig slurry samples during aerobic decomposition. The blue lines represent observations, corrected for background emissions from the soil, while the red lines represent 95% confidence limits of model fits.
(PDF)

S1 Table Summary of in-house storage conditions at the time of sampling. Where possible, information about time of last emptying was recorded for calculation of collection period (continued on next page).
(PDF)

S2 Table Selected properties of the slurry materials collected for this study. (PDF)

S3 Table Dry matter (%) in slurry delivered to Thorsø Biogas plant during 2014. For information about farms, please refer to S1 Table. Data were obtained from the biogas plant manager, Anders Nedergaard.
(PDF)

S4 Table Amounts of residual volatile solids (VS) in two pools (VSd = easily degradableThe 95% confidence intervals represent the confidence limits of observed storage temperatures for pig and cattle slurry. **Given that excretal returns VS; VSnd = "non-degradable" VS), with daily time steps.** The proportions of CH4 and CO2 emitted are unknown. Here residual VS were calculated assuming CH4-C/(CH4-C + CO2-C) ratios of 0.05, 0.1, 0.3 and 0.5, respectively. The 95% confidence intervals represent the confidence limits of observed storage temperatures for pig and cattle slurry. Given that excretal returns are added each day, The best estimate of residual VS at sampling is the average value of the 15-day (pig slurry) or 30-day (cattle slurry) storage period, and these values, with C.I., are shown in Fig 3.
(PDF)
