# Supplementary material for: Estimation of Methane Emissions from Slurry Pits below Pig and Cattle Confinements
Source: PLoS One. 2016 Aug 16;11(8):e0160968. doi: 10.1371/journal.pone.0160968 (PMC4986936; doi:10.1371/journal.pone.0160968)
Supplement: S1 Fig — (PDF) [file pone.0160968.s001.pdf]

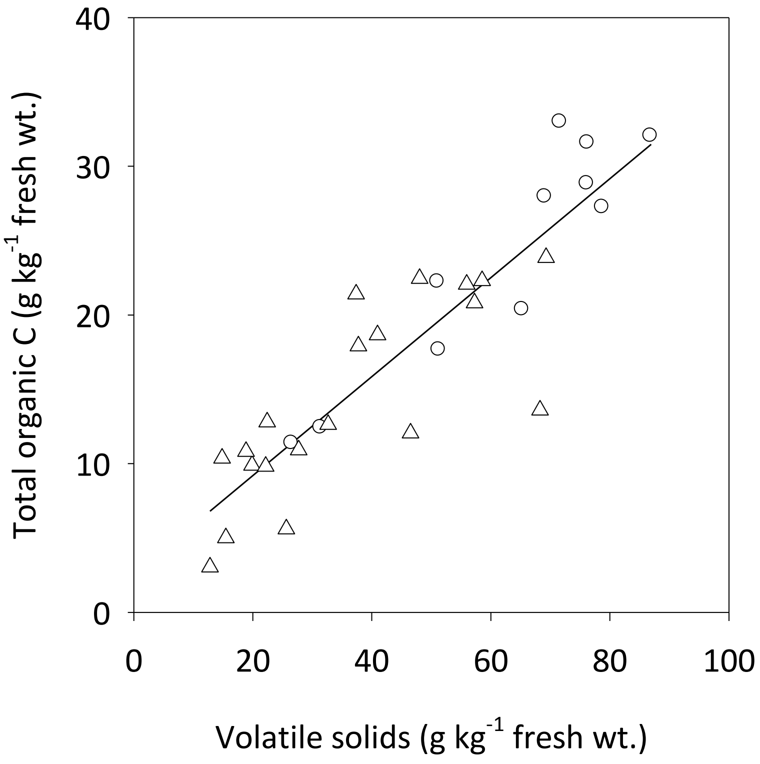

S1 Fig. Relationship between volatile solids in cattle (circles) and pig (triangles) slurry materials and TOC.
