# Supplementary material for: Estimation of Methane Emissions from Slurry Pits below Pig and Cattle Confinements
Source: PLoS One. 2016 Aug 16;11(8):e0160968. doi: 10.1371/journal.pone.0160968 (PMC4986936; doi:10.1371/journal.pone.0160968)
Supplement: S3 Fig — The blue lines represent observations, corrected for background emissions from the soil, while the red lines represent 95% confidence limits of model fits. (PDF) [file pone.0160968.s003.pdf]

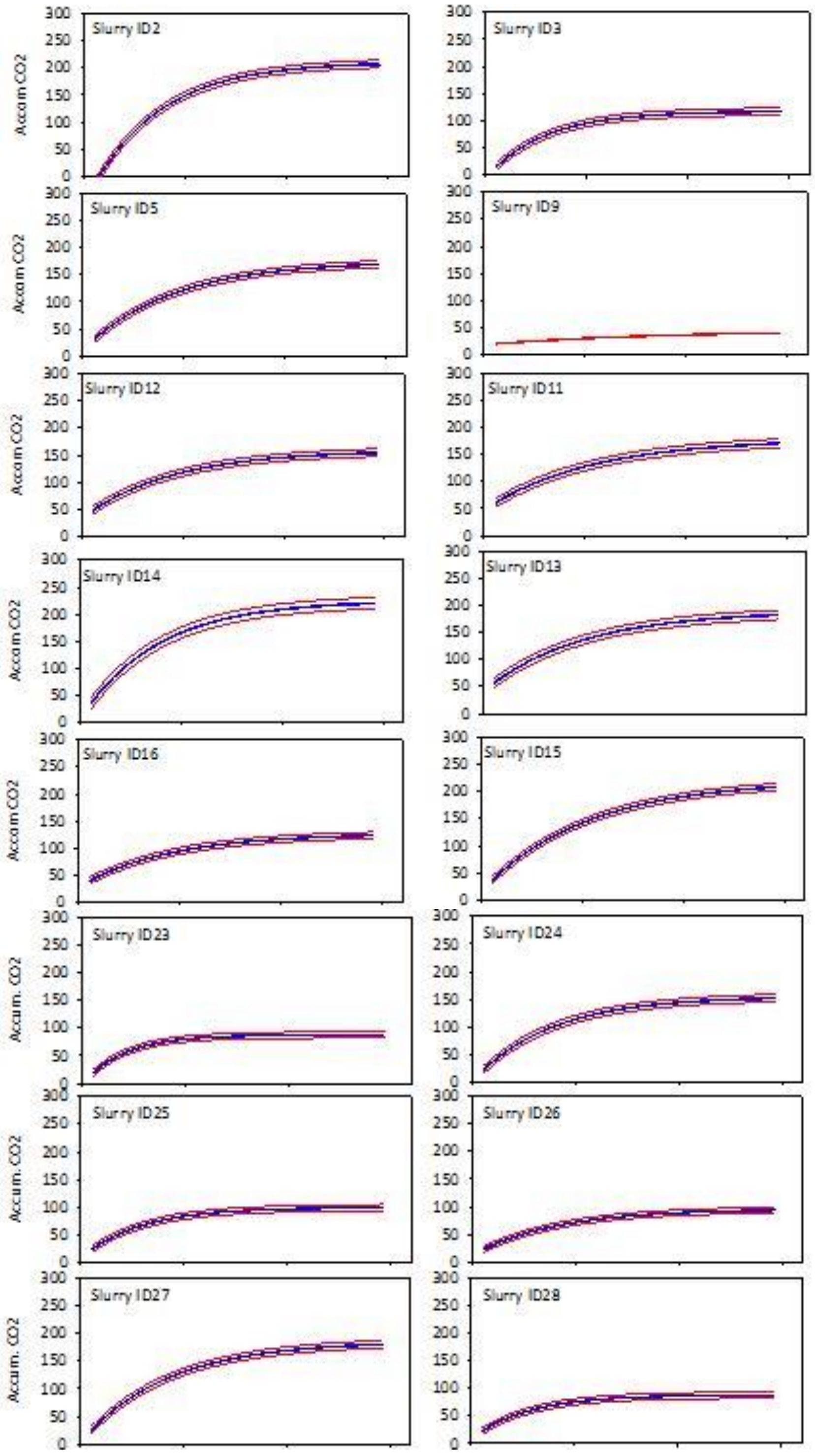

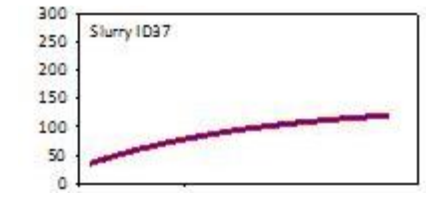

S3 Fig. Evolution of CO_2-C from pig slurry samples during aerobic decomposition. The blue lines represent observations, corrected for background emissions from the soil, while the red lines represent 95% confidence limits of model fits.
