# Supplementary material for: Estimation of Methane Emissions from Slurry Pits below Pig and Cattle Confinements
Source: PLoS One. 2016 Aug 16;11(8):e0160968. doi: 10.1371/journal.pone.0160968 (PMC4986936; doi:10.1371/journal.pone.0160968)
Supplement: S1 Table — Where possible, information about time of last emptying was recorded for calculation of collection period (continued on next page). (PDF) [file pone.0160968.s004.pdf]

S1 Table. Summary of in-house storage conditions at the time of sampling. Where possible, information about time of last emptying was recorded for calculation of collection period (continued on next page).

Sample ID	Farm No	Animal category	Depth of pit (cm)	Slurry height (cm)	Collection period (d)	Air temperature (°C)	Slurry temperature (°C)
1	G3	Piglets	60	5	4	17.5	18.1
2	G3	Piglets	60	30	29	15.5	16.9
3	G3	Piglets	60	25	14	16.4	18.4
4	G3	Piglets	60	Empty	- [§]	-	-
5	G3	Finishers	50	25	8	14.5	14.8
6	G3	farrowing sows	90	20	0	9.9	17.6
7	G3	farrowing sows	40	20	14	14.5	15.2
8	G3	farrowing sows	40	20	7	15.3	17.5
9	G3	farrowing sows	40	35	14	15.2	18.4
10	G3	farrowing sows	40	20	14	15.2	17.5
11	G4	finishers	60	10	1	17.3	19.4
12	G4	finishers	60	10	5	17.7	20.1
13	G4	finishers	60	35	>12	21.7	22.3
14	G4	finishers	60	10	>12	17.4	20.2
15	G4	finishers	60	30	>12	15.2	17.7
16	G4	finishers	60	25	>12	18.2	21.0
17	G6	dairy	40	nearly full	-	3.2	5.5
18	G6	dairy	120	nearly full	2	3.2	9.1
19	G2	dairy	120	nearly full	2	7.7	9.4
20	G5	dairy	120	nearly full	2	6.1	10.5
21	G5	dairy	120	nearly full	2	4.7	9.3
22	G1	dairy	120	nearly full	2	4.7	12.3
23	G4	finishers	60	25	15	16.7	20.6
24	G4	finishers	60	20	19	17.3	21.4
25	G4	finishers	60	15	>12	20.3	22.0
26	G4	finishers	60	20	15	18.4	20.0
27	G4	finishers	60	25	6	16.0	18.1
28	G4	finishers	60	20	8	18.4	18.6
29	G6	dairy	40	nearly full	7	4.5	7.4
30	G6	dairy	120	nearly full	4	4.5	8.8
31	G2	dairy	120	nearly full	-	7.2	9.7
32	G5	dairy	120	nearly full	3	5.8	9.0
33	G5	dairy	120	nearly full	3	5.8	10.7
34	G1	dairy	120	nearly full	-	4.7	16.0
35	G7	farrowing sows	40	20	13	15.6	17.4

36	G7	farrowing sows	40	30	13	15.6	18.9
37	G7	farrowing sows	55	25	13	13.8	19.4
38	G7	farrowing sows	- §	5	7	14.6	16.4
39	G7	farrowing sows	220	120	-	-	16.7

§ No information available.
