# Supplementary material for: Estimation of Methane Emissions from Slurry Pits below Pig and Cattle Confinements
Source: PLoS One. 2016 Aug 16;11(8):e0160968. doi: 10.1371/journal.pone.0160968 (PMC4986936; doi:10.1371/journal.pone.0160968)
Supplement: S2 Table — (PDF) [file pone.0160968.s005.pdf]

S2 Table. Selected properties of the slurry materials collected for this study.

Sample ID	Farm No	Animal category	DM	VS	TOC	Conductivity	pH
			(%)	(g/kg)	(g/kg)	(mS/cm)	
1	G3	Pig	15.5	103	#I/T	15.6	6.3
2	G3	Pig	8.6	69	24	16	6.3
3	G3	Pig	2.8	20	10	41.5	6.8
4	G3	Pig	Empty	- §	-	-	-
5	G3	Pig	4.9	38	18	59.5	7
6	G3	Pig	12.8	98	#I/T	13.9	6.2
7	G3	Pig	1.5	8	#I/T	64	7.9
8	G3	Pig	0.9	5	#I/T	17.6	8.4
9	G3	Pig	1.9	13	3	16.6	7.8
10	G3	Pig	2.3	15	5	17.1	7.5
11	G4	Pig	6.1	41	19	78	7.5
12	G4	Pig	4.1	28	11	76.8	7.4
13	G4	Pig	5	33	13	84.7	7.6
14	G4	Pig	7.9	56	22	76.6	7.3
15	G4	Pig	8	59	22	65.5	7.2
16	G4	Pig	3.5	22	10	66.8	7.1
17	G6	Cattle	9.7	72	33	17	6.9
18	G6	Cattle	9.5	76	32	10.8	7.1
19	G2	Cattle	6.4	51	22	10.2	7.4
20	G5	Cattle	4.1	31	12	8.7	7.3
21	G5	Cattle	11	87	32	12	7.8
22	G1	Cattle	20.7	93	#I/T	10.2	6.7
23	G4	Pig	2.7	15	10	18.5	7.3
24	G4	Pig	6.8	48	22	15.4	6.9
25	G4	Pig	3.5	19	11	19.1	7.2
26	G4	Pig	6.7	37	21	13.3	7.3
27	G4	Pig	8.1	57	21	13.5	7.2
28	G4	Pig	3.7	22	13	14.9	7.1
29	G6	Cattle	11.2	76	29	7.7	6.9
30	G6	Cattle	8.7	69	28	8.2	7
31	G2	Cattle	6.6	51	18	8.4	7.3
32	G5	Cattle	3.5	27	11	6.1	7.5
33	G5	Cattle	10.3	79	27	11	8.3
34	G1	Cattle	7.8	65	20	6	6.7
35	G7	Pig	15.3	93	#I/T	0	7.4
36	G7	Pig	8.6	68	14	8.6	7.6
37	G7	Pig	6.5	47	12	11.3	8
38	G7	Pig	3.3	26	6	6.5	8
39	G7	Pig	0.9	4	3	8.1	7.9

§ No information available.
