# Supplementary material for: Estimation of Methane Emissions from Slurry Pits below Pig and Cattle Confinements
Source: PLoS One. 2016 Aug 16;11(8):e0160968. doi: 10.1371/journal.pone.0160968 (PMC4986936; doi:10.1371/journal.pone.0160968)
Supplement: S3 Table — For information about farms, please refer to S1 Table. Data were obtained from the biogas plant manager, Anders Nedergaard. (PDF) [file pone.0160968.s006.pdf]

S3 Table. Dry matter (%) in slurry delivered to Thorsø Biogas plant during 2014. For information about farms, please refer to Table S1. Data were obtained from the biogas plant manager, Anders Nedergaard.

**Farm ID** 1)	**Animal category**	**Housing system**	**DM (%)**		
			Avg	min	max
G1	Dairy cattle	Cubicles	6.5	6	6.9
G2	Dairy cattle	Cubicles	5.6	4.8	6.4
G3	Finishing pigs	Partly slatted	4.1	3.3	5
G4	Finishing pigs	Partly slatted	5.2	4.6	6.3
G5	Dairy cattle	Cubicles	6.5	5.3	8.6
G6	Dairy cattle	Cubicles	9.2	9.2	9.2
G7	Farrowing sows	Loose, indiv confinement	3.5	1	7

1) Geographical coordinates: G1 - 56°21'10" N, 9°48'10" E; G2 - 56°18'02" N, 9°43'42" E; G3 - 56°20'13" N, 9°53'13" E; G4 - 56°21'06" N, 9°45'26" E; G5 - 56°21'29" N, 9°52'12" E; G6 - 56°16'11" N, 9°45'24" E; G7 - 56°21'02" N, 9°49'06" E.
