# Supplementary material for: Estimation of Methane Emissions from Slurry Pits below Pig and Cattle Confinements
Source: PLoS One. 2016 Aug 16;11(8):e0160968. doi: 10.1371/journal.pone.0160968 (PMC4986936; doi:10.1371/journal.pone.0160968)
Supplement: S4 Table — Given that excretal returns VS; VSnd = "non-degradable" VS), with daily time steps. The proportions of CH4 and CO2 emitted are unknown. Here residual VS were calculated assuming CH4-C/(CH4-C + CO2-C) ratios of 0.05, 0.1, 0.3 and 0.5, respectively. The 95% confidence intervals represent the confidence limits of observed storage temperatures for pig and cattle slurry. Given that excretal returns are added each day, The best estimate of residual VS at sampling is the average value of the 15-day (pig slurry) or 30-day (cattle slurry) storage period, and these values, with C.I., are shown in Fig 3. (PDF) [file pone.0160968.s007.pdf]

S4 Table. Amounts of residual volatile solids (VS) in two pools (VSd = easily degradableThe 95% confidence intervals represent the confidence limits of observed storage temperatures for pig and cattle slurry. Given that excretal returns VS; VSnd = "non-degradable" VS), with daily time steps. The proportions of CH4 and CO2 emitted are unknown. Here residual VS were calculated assuming CH4-C/(CH4-C + CO2-C) ratios of 0.05, 0.1, 0.3 and 0.5, respectively. The 95% confidence intervals represent the confidence limits of observed storage temperatures for pig and cattle slurry. Given that excretal returns are added each day, The best estimate of residual VS at sampling is the average value of the 15-day (pig slurry) or 30-day (cattle slurry) storage period, and these values, with C.I., are shown in Fig. 3.

Slurry type:	Pig slurry																							
H-C/(CH4-C + CO2-C) ratio:	0.05						0.1						0.3						0.6					
Volatile solids remaining:	VSd			VSnd			VSd			VSnd			VSd			VSnd			VSd			VSnd		
Days of storage	Avg	-95% C.I.	+95% C.I.	Avg	-95% C.I.	+95% C.I.	Avg	-95% C.I.	+95% C.I.	Avg	-95% C.I.	+95% C.I.	Avg	-95% C.I.	+95% C.I.	Avg	-95% C.I.	+95% C.I.	Avg	-95% C.I.	+95% C.I.	Avg	-95% C.I.	+95% C.I.
1	0.890	0.890	0.890	0.110	0.110	0.110	0.890	0.890	0.890	0.110	0.110	0.110	0.890	0.890	0.890	0.110	0.110	0.110	0.890	0.890	0.890	0.110	0.110	0.110
2	0.802	0.830	0.746	0.110	0.110	0.110	0.846	0.860	0.818	0.110	0.110	0.110	0.875	0.880	0.866	0.110	0.110	0.110	0.883	0.885	0.878	0.110	0.110	0.110
3	0.722	0.775	0.625	0.110	0.110	0.110	0.804	0.831	0.752	0.110	0.110	0.110	0.861	0.870	0.843	0.110	0.110	0.110	0.875	0.880	0.866	0.110	0.110	0.110
4	0.651	0.723	0.524	0.110	0.110	0.109	0.764	0.804	0.691	0.110	0.110	0.110	0.847	0.861	0.820	0.110	0.110	0.110	0.868	0.875	0.854	0.110	0.110	0.110
5	0.586	0.674	0.439	0.110	0.110	0.109	0.726	0.777	0.635	0.110	0.110	0.110	0.833	0.851	0.798	0.110	0.110	0.110	0.861	0.870	0.843	0.110	0.110	0.110
6	0.528	0.629	0.368	0.109	0.110	0.109	0.690	0.751	0.583	0.110	0.110	0.110	0.819	0.841	0.776	0.110	0.110	0.110	0.854	0.865	0.832	0.110	0.110	0.110
7	0.476	0.587	0.308	0.109	0.110	0.109	0.656	0.725	0.536	0.110	0.110	0.109	0.805	0.832	0.755	0.110	0.110	0.110	0.847	0.861	0.820	0.110	0.110	0.110
8	0.429	0.548	0.258	0.109	0.109	0.109	0.624	0.701	0.493	0.110	0.110	0.109	0.792	0.823	0.735	0.110	0.110	0.110	0.840	0.856	0.809	0.110	0.110	0.110
9	0.386	0.511	0.216	0.109	0.109	0.109	0.593	0.678	0.453	0.110	0.110	0.109	0.779	0.814	0.715	0.110	0.110	0.110	0.833	0.851	0.798	0.110	0.110	0.110
10	0.348	0.477	0.181	0.109	0.109	0.108	0.563	0.655	0.416	0.110	0.110	0.109	0.766	0.804	0.696	0.110	0.110	0.110	0.826	0.846	0.788	0.110	0.110	0.110
11	0.313	0.445	0.152	0.109	0.109	0.108	0.535	0.633	0.382	0.109	0.110	0.109	0.753	0.795	0.677	0.110	0.110	0.110	0.819	0.842	0.777	0.110	0.110	0.110
12	0.282	0.415	0.127	0.109	0.109	0.108	0.509	0.612	0.351	0.109	0.110	0.109	0.741	0.787	0.659	0.110	0.110	0.110	0.812	0.837	0.766	0.110	0.110	0.110
13	0.254	0.387	0.107	0.109	0.109	0.108	0.484	0.591	0.323	0.109	0.110	0.109	0.729	0.778	0.641	0.110	0.110	0.110	0.806	0.832	0.756	0.110	0.110	0.110
14	0.229	0.361	0.089	0.109	0.109	0.108	0.460	0.572	0.297	0.109	0.110	0.109	0.717	0.769	0.624	0.110	0.110	0.110	0.799	0.828	0.746	0.110	0.110	0.110
15	0.206	0.337	0.075	0.108	0.109	0.108	0.437	0.552	0.273	0.109	0.109	0.109	0.705	0.761	0.607	0.110	0.110	0.110	0.792	0.823	0.736	0.110	0.110	0.110

Slurry type:	Cattle slurry																							
H-C/(CH4-C + CO2-C) ratio:	0.05						0.1						0.3						0.6					
Volatile solids remaining:	VSd			VSnd			VSd			VSnd			VSd			VSnd			VSd			VSnd		
Days of storage	Avg	-95% C.I.	+95% C.I.	Avg	-95% C.I.	+95% C.I.	Avg	-95% C.I.	+95% C.I.	Avg	-95% C.I.	+95% C.I.	Avg	-95% C.I.	+95% C.I.	Avg	-95% C.I.	+95% C.I.	Avg	-95% C.I.	+95% C.I.	Avg	-95% C.I.	+95% C.I.
1	0.460	0.460	0.460	0.540	0.540	0.540	0.460	0.460	0.460	0.540	0.540	0.540	0.460	0.460	0.460	0.540	0.540	0.540	0.460	0.460	0.460	0.540	0.540	0.540
2	0.417	0.436	0.374	0.539	0.540	0.539	0.438	0.448	0.417	0.540	0.540	0.539	0.453	0.456	0.446	0.540	0.540	0.540	0.456	0.458	0.453	0.540	0.540	0.540
3	0.378	0.413	0.304	0.539	0.539	0.538	0.418	0.436	0.378	0.539	0.540	0.539	0.446	0.452	0.432	0.540	0.540	0.540	0.453	0.456	0.446	0.540	0.540	0.540
4	0.342	0.392	0.247	0.538	0.539	0.537	0.398	0.425	0.342	0.539	0.540	0.538	0.439	0.448	0.418	0.540	0.540	0.539	0.449	0.454	0.439	0.540	0.540	0.540
5	0.310	0.372	0.200	0.538	0.539	0.536	0.379	0.414	0.310	0.539	0.539	0.538	0.432	0.444	0.405	0.540	0.540	0.539	0.446	0.452	0.432	0.540	0.540	0.540
6	0.281	0.352	0.163	0.537	0.539	0.535	0.362	0.403	0.281	0.539	0.539	0.537	0.425	0.440	0.392	0.540	0.540	0.539	0.442	0.450	0.425	0.540	0.540	0.540
7	0.254	0.334	0.132	0.537	0.538	0.534	0.345	0.393	0.255	0.538	0.539	0.537	0.418	0.437	0.380	0.539	0.540	0.539	0.439	0.448	0.419	0.540	0.540	0.539
8	0.231	0.317	0.107	0.536	0.538	0.533	0.328	0.383	0.231	0.538	0.539	0.536	0.412	0.433	0.368	0.539	0.540	0.539	0.435	0.446	0.412	0.540	0.540	0.539
9	0.209	0.300	0.087	0.536	0.538	0.532	0.313	0.373	0.209	0.538	0.539	0.536	0.405	0.429	0.357	0.539	0.540	0.539	0.432	0.444	0.406	0.540	0.540	0.539
10	0.189	0.285	0.071	0.535	0.537	0.531	0.298	0.363	0.190	0.538	0.539	0.535	0.399	0.425	0.346	0.539	0.540	0.538	0.429	0.442	0.399	0.540	0.540	0.539
11	0.172	0.270	0.058	0.535	0.537	0.530	0.284	0.354	0.172	0.537	0.539	0.535	0.393	0.422	0.335	0.539	0.540	0.538	0.425	0.440	0.393	0.540	0.540	0.539
12	0.155	0.256	0.047	0.534	0.537	0.529	0.271	0.344	0.156	0.537	0.538	0.534	0.387	0.418	0.324	0.539	0.539	0.538	0.422	0.439	0.387	0.540	0.540	0.539
13	0.141	0.242	0.038	0.534	0.537	0.528	0.258	0.335	0.141	0.537	0.538	0.534	0.381	0.414	0.314	0.539	0.539	0.538	0.419	0.437	0.381	0.539	0.540	0.539
14	0.128	0.230	0.031	0.533	0.536	0.527	0.246	0.327	0.128	0.537	0.538	0.533	0.375	0.411	0.304	0.539	0.539	0.538	0.415	0.435	0.375	0.539	0.540	0.539
15	0.116	0.218	0.025	0.533	0.536	0.526	0.235	0.318	0.116	0.536	0.538	0.533	0.369	0.407	0.295	0.539	0.539	0.538	0.412	0.433	0.369	0.539	0.540	0.539
16	0.105	0.207	0.020	0.532	0.536	0.525	0.224	0.310	0.105	0.536	0.538	0.532	0.363	0.404	0.286	0.539	0.539	0.537	0.409	0.431	0.363	0.539	0.540	0.539
17	0.095	0.196	0.017	0.532	0.536	0.524	0.213	0.302	0.095	0.536	0.538	0.532	0.357	0.400	0.277	0.539	0.539	0.537	0.406	0.429	0.358	0.539	0.540	0.539
18	0.086	0.186	0.013	0.531	0.535	0.523	0.203	0.294	0.086	0.536	0.538	0.531	0.352	0.397	0.268	0.539	0.539	0.537	0.402	0.427	0.352	0.539	0.540	0.539
19	0.078	0.176	0.011	0.531	0.535	0.522	0.193	0.286	0.078	0.535	0.537	0.531	0.346	0.393	0.260	0.538	0.539	0.537	0.399	0.425	0.346	0.539	0.540	0.538
20	0.189	0.285	0.071	0.535	0.537	0.531	0.298	0.363	0.190	0.538	0.539	0.535	0.399	0.425	0.346	0.539	0.540	0.538	0.429	0.442	0.399	0.540	0.540	0.539
21	0.172	0.270	0.058	0.535	0.537	0.530	0.284	0.354	0.172	0.537	0.539	0.535	0.393	0.422	0.335	0.539	0.540	0.538	0.425	0.440	0.393	0.540	0.540	0.539
22	0.155	0.256	0.047	0.534	0.537	0.529	0.271	0.344	0.156	0.537	0.538	0.534	0.387	0.418	0.324	0.539	0.539	0.538	0.422	0.439	0.387	0.540	0.540	0.539
23	0.141	0.242	0.038	0.534	0.537	0.528	0.258	0.335	0.141	0.537	0.538	0.534	0.381	0.414	0.314	0.539	0.539	0.538	0.419	0.437	0.381	0.539	0.540	0.539
24	0.128	0.230	0.031	0.533	0.536	0.527	0.246	0.327	0.128	0.537	0.538	0.533	0.375	0.411	0.304	0.539	0.539	0.538	0.415	0.435	0.375	0.539	0.540	0.539
25	0.116	0.218	0.025	0.533	0.536	0.526	0.235	0.318	0.116	0.536	0.538	0.533	0.369	0.407	0.295	0.539	0.539	0.538	0.412	0.433	0.369	0.539	0.540	0.539
26	0.281	0.352	0.163	0.537	0.539	0.535	0.362	0.403	0.281	0.539	0.539	0.537	0.425	0.440	0.392	0.540	0.540	0.539	0.442	0.450	0.425	0.540	0.540	0.540
27	0.254	0.334	0.132	0.537	0.538	0.534	0.345	0.393	0.255	0.538	0.539	0.537	0.418	0.437	0.380	0.539	0.540	0.539	0.439	0.448	0.419	0.540	0.540	0.539
28	0.231	0.317	0.107	0.536	0.538	0.533	0.328	0.383	0.231	0.538	0.539	0.536	0.412	0.433	0.368	0.539	0.540	0.539	0.435	0.446	0.412	0.540	0.540	0.539
29	0.209	0.300	0.087	0.536	0.538	0.532	0.313	0.373	0.209	0.538	0.539	0.536	0.405	0.429	0.357	0.539	0.540	0.539	0.432	0.444	0.406	0.540	0.540	0.539
30	0.189	0.285	0.071	0.535	0.537	0.531	0.298	0.363	0.190	0.538	0.539	0.535	0.399	0.425	0.346	0.539	0.540	0.538	0.429	0.442	0.399	0.540	0.540	0.539
